# Ultra-broadband Nonlinear Saturable Absorption for Two-dimensional Bi_2_Te_*x*_Se_*3−x*_ Nanosheets

**DOI:** 10.1038/srep33070

**Published:** 2016-09-09

**Authors:** Yingwei Wang, Sheng Liu, Jian Yuan, Peng Wang, Jiazhang Chen, Jianbo Li, Si Xiao, Qiaoliang Bao, Yongli Gao, Jun He

**Affiliations:** 1 School of Physics and Electronics, Hunan Key Laboratory for Super-micro structure and Ultrafast Process, Central South University, 932 South Lushan Road, Changsha, Hunan 410083, P. R. China; 2 Institute of Functional Nano and Soft Materials (FUNSOM), Jiangsu Key Laboratory for Carbon-Based Functional Materials and Devices, and Collaborative Innovation Center of Suzhou Nano Science and Technology, Soochow University, Suzhou 215123, P. R. China; 3 Institute of Mathematics and Physics, Central South University of Forestry and Technology, Changsha 410004, China; 4 Department of Physics and Astronomy, University of Rochester, Rochester, NewYork 14627, United States

## Abstract

We report the ultra-broadband nonlinear optical (NLO) response of Bi_2_Te_*x*_Se_*3−x*_ nanosheets produced by a facile solvothermal method. Our result show that the extracted basic optical nonlinearity parameters of Bi_2_Te_*x*_Se_*3−x*_ nanosheets, α_NL_, Imχ^(3)^, and FOM reach ~10^4^ cm/GW, ~10^−8^ esu and ~10^−13^ esu cm, respectively, which are several orders of magnitude larger than those of bulk dielectrics. We further observed the excitation intensity dependence of the NLO absorption coefficient and the NLO response sensitivity. The mechanisms of those phenomena were proposed based on physical model. The wavelength dependence of the NLO response of Bi_2_Te_*x*_Se_*3−x*_ nanosheets was investigated, and we determined that the Bi_2_Te_*x*_Se_*3−x*_ nanosheets possess an ultra-broadband nonlinear saturable absorption property covering a range from the visible to the near-infrared band, with the NLO absorption insensitive to the excitation wavelength. This work provide fundamental and systematic insight into the NLO response of Bi_2_Te_*x*_Se_*3−x*_ nanosheets and support their application in photonic devices in the future.

Topological insulators (TIs) are electronic materials that exhibit an insulation state for their bulk but a conduction state on their edge and surface^1^,^2^,^3^,^4^. In recent years, significant efforts have been devoted to controlling the crystal morphology of TIs^5^,^6^,^7^, extending their applications in the electronics and optoelectronics fields^8^,^9^,^10^, and improving their performance in thermoelectric devices^11^,^12^.

Owing to the strong light-matter interactions in two-dimensional (2D) materials, the linear optical properties of the Bi_2_Te_*x*_Se_*3−x*_ family have been intensely studied in previous works^13^,^14^,^15^. Meanwhile, inspired by the progress of NLO applications of TI materials^16^,^17^,^18^,^19^,^20^, NLO research into TIs has attracted a great deal of interest. Zhang *et al*.^21^,^22^,^23^ demonstrated a series realization of ultra-short pulse generation based on a Bi_2_Se_3_ saturable absorber. Subsequently, similar work on other members of the Bi_2_Te_*x*_Se_*3−x*_ family, i.e., Bi_2_Te_3_, Bi_2_TeSe_2_, and Bi_2_Te_2_Se^24^,^25^,^26^,^27^,^28^, was reported. However, those works generally focused on the modulation depth, saturation intensity and nonsaturable loss. Although those are necessary indicators for the application of saturable absorbers, investigation into the basic NLO parameters, such as the NLO absorption coefficient α_NL_ and third-order NLO susceptibility Imχ^(3)^, has been limited. Those basic coefficients are important optical parameters for 2D TIs, which determine the performance of 2D TIs-based nanophotonic devices.

In this work, using a high-yield facile solvothermal method, we synthesized large-scale and high-quality Bi_2_Te_*x*_Se_*3−x*_ nanosheets and verified the process using material characterization. For experimental investigation of NLO response, self-phase modulation^29^,^30^,^31^,^32^ and Z-scan measurement^20^,^33^,^34^ are the most popular experimental methods, and we chose the latter. Significant saturable absorption was observed at all of the excitation wavelengths (532 nm, 800 nm, 1050 nm and 1550 nm), which indicated their ultra-broadband saturable absorption characteristics. Based on the NLO theory model, we extracted the NLO absorption coefficient α_NL_, third-order NLO susceptibility Imχ^(3)^, figure of merit (FOM) for the third-order optical nonlinearity, and saturation intensity *I*_s_ from the Z-scan result. The excellent NLO response, i.e., α_NL_ ~ 10^4^ cm/GW, Imχ^(3)^ ~ 10^−9^ esu, FOM ~10^−13^ esu cm and *I*_s_ ~ 10^9^ W/cm^2^, implies that the Bi_2_Te_*x*_Se_*3−x*_ nanosheets have the potential for application in nanophotonic devices. The results for the different excitation wavelengths demonstrated that the NLO response is independent of the excitation wavelength. Additionally, it was found that atom-doped Bi_2_Te_*x*_Se_*3−x*_nanosheets (Bi_2_TeSe_2_, Bi_2_Te_2_Se) showed a more sensitive nonlinear absorption response to photoexcitation.

## Materials and Methods

### Synthesis of Bi_2_Te_*x*_Se_*3−x*_nanosheets

The precursor materials for the solvothermal synthesis of Bi_2_Te_*x*_Se_*3−x*_ nanosheets were bismuth nitrate (Bi(NO_3_)_3_), bismuth oxide (Bi_2_O_3_), selenium oxide (SeO_2_) and tellurium oxide (TeO_2_), all of which were purchased from Alfa Aesar. Polyvinylpyrrolidone (PVP, K30) was purchased from TCI, and sodium hydroxide (NaOH) and ethylene glycol (EG) were acquired from Shanghai Chemical Reagent Co. (Shanghai, China). All of the chemicals were used as received without further purification.

The Bi_2_Te_*x*_Se_*3−x*_ nanosheets, i.e., Bi_2_Se_3_, Bi_2_Te_3_, Bi_2_TeSe_2_, Bi_2_Te_2_Se, were synthesized by the same procedures as described in our previous work, ref. 5. Based on the solvothermal method, we produced high-quality Bi_2_Te_*x*_Se_*3−x*_nanosheets with a regular hexagonal morphology and good crystallinity.

### Material characterization

The morphology of the graphene-Bi_2_Te_3_ heterostructure was investigated by scanning electron microscopy (SEM) (FEI Quanta 200 FEG, acceleration voltage: 5–30 kV) and transmission electron microscopy (TEM) (HRTEM, FEI Tecnai G2 F20 STWIN, FEI). The topography and thickness of the as-produced samples were determined by atomic force microscopy (AFM) (Agilent 5500). The optical nonlinearity of the sample was investigated by a conventional Z-scan technique. A femtosecond laser pulse was produced by an optical parametric amplifier (TOPAS, USF-UV2) that was pumped by a Ti: Sapphire regenerative amplifier system (Spectra-Physics, Spitfire ACE-35F-2KXP, Maitai SP and Empower 30) with a pulse duration of 35 fs and a pulse repetition rate of 2 kHz. The laser beam was focused by a lens with a focus length of 150 mm. A computer-controlled translation stage was employed to move the sample along the propagation direction (*z*-axis) of the laser pulses, and the transmitted pulse energies were probed by a detector (OPHIR, PD300R-IR).

## Results and Discussion

**Figure 1:**
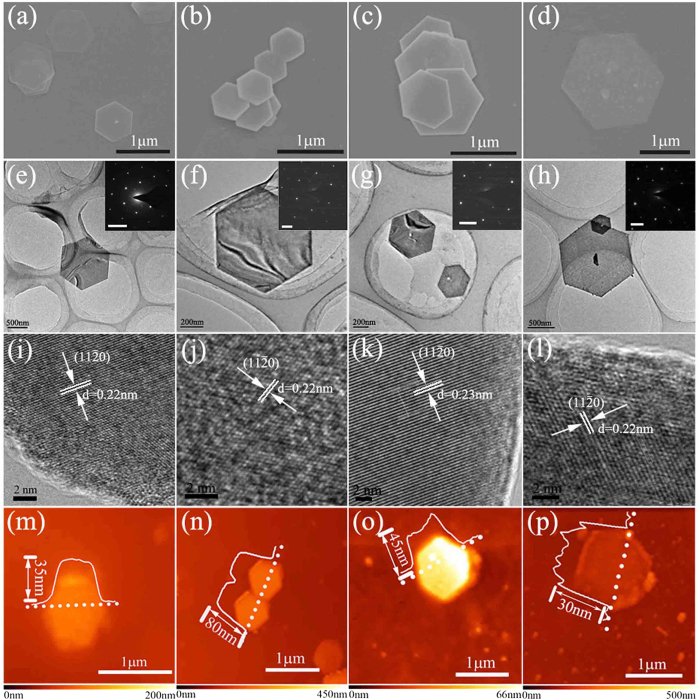
Characterization of Bi_2_Te_*x*_Se_*3−x*_ nanosheets: (**a**–**d**) Scanning electron microscopy (SEM), (**e**–**h**) transmission electron microscopy (TEM), (**i**–**l**) high-resolution transmission electron microscopy (HRTEM) and (**m**–**n**) atomic force microscopy (AFM) images of Bi_2_Te_3_, Bi_2_Te_2_Se, Bi_2_TeSe_2_ and Bi_2_Se_3_. The insets of (**e**–**h**) are the selected area electron diffraction (SAED) patterns of the nanosheets.

The morphology of the as-prepared Bi_2_Te_*x*_Se_*3−x*_ nanosheets was characterized by SEM, AFM and TEM. As shown in Fig. 1(a–d), we find that the hexagonal nanosheets are largely distributed on the substrate in high yield. The excellent crystallinity can be well defined by the regular shape and sharp edges. The average size of the platelets is from 400 nm to 5 μm.

Figure 1(e–h) depict the TEM images of the Bi_2_Te_*x*_Se_*3−x*_ nanosheets. It can be seen that the thickness is symmetrical, with a relatively uniform contrast. The selected area electron diffraction (SAED) patterns shown as insets indicate the single-crystalline nature of these nanosheets. Figure 1(i–l) depict high-resolution transmission electron microscopy (HRTEM) images. Figure 1(i) clearly presents that the lattice space of Bi_2_Te_3_ is approximately 0.22 nm, corresponding to the spacing of the (110) planes of the rhombohedral phase. In Fig. 1(j–l), the spacings of 0.22 nm, 0.23 nm, and 0.22 nm are consistent with the lattice spaces of Bi_2_Te_2_Se, Bi_2_TeSe_2_, and Bi_2_Se_3_, respectively. It is implied that the nanosheets are well-crystallized with few atomic defects. The AFM is used to determine the sample thickness, as shown in Fig. 1(m–p). By the analysis of the height profile (across the dotted line inset of the AFM image), the average thickness of the sample is determined to be 35 nm, 80 nm, 45 nm and 30 nm for Bi_2_Te_3_, Bi_2_Te_2_Se, Bi_2_TeSe_2_ and Bi_2_Se_3_, respectively. The material characterization of the Bi_2_Te_*x*_Se_*3−x*_nanosheets indicates that all our samples possess a similar submicron-scale morphology, nanoscale thickness and excellent crystallinity.

**Figure 2:**
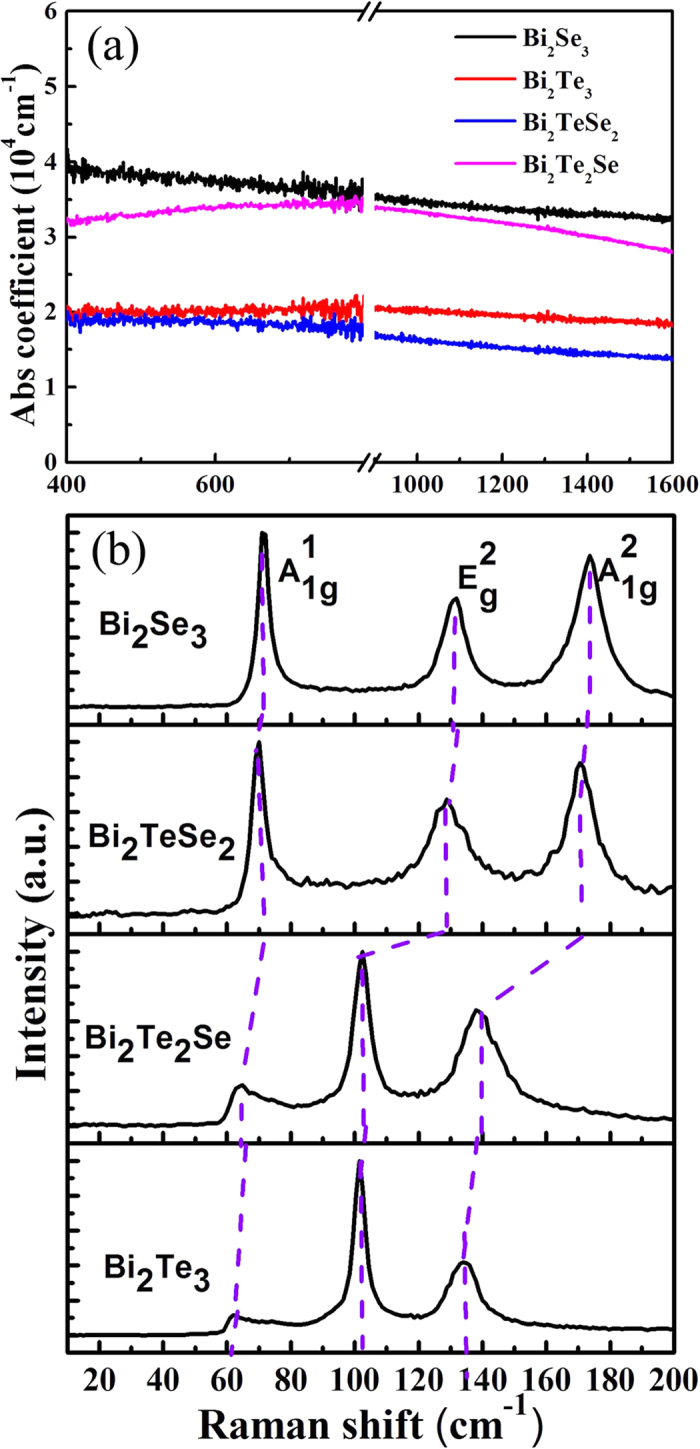
(**a**) Absorption spectra and (**b**) Raman spectra of Bi_2_Te_*x*_Se_*3−x*_ nanosheets, taken with an excitation laser wavelength of 633 nm.

The linear absorption spectrum of the Bi_2_Te_*x*_Se_*3−x*_ nanosheets is shown in Fig. 2(a). An ultra-broadband linear optical absorption is observed, which is ascribed to the gapless surface state and narrow bulk state gap. Furthermore, we extract the linear absorption coefficient α_0_ from the absorption spectrum of the sample. The absorption coefficient of the Bi_2_Te_*x*_Se_*3−x*_nanosheets is determined to be ~10^4^ cm^−1^.

The Raman spectrum of the Bi_2_Te_*x*_Se_*3−x*_ nanosheets is shown in Fig. 2(b) and was obtained by a laser excitation wavelength of 633 nm. It is observed that the typical three main Raman peaks corresponding to 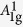, 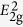 and 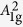 are located in low frequency range from 30 to 200 cm^−1^ for all compounds. For the Bi_2_Te_3_ nanosheets, the three Raman peaksappear at 62 cm^−1^, 102 cm^−1^ and 137 cm^−1^, respectively. With more Se incorporation and the replacement of Te, the vibrational modes of the Bi_2_Te_2_Se nanosheets are slightly red-shifted, i.e., 65 cm^−1^, 102 cm^−1^ and 138 cm^−1^, compared with those of the Bi_2_Te_3_ nanosheets. For Bi_2_TeSe_2_ and Bi_2_Se_3_, significant red-shifts exist for the three main peaks, i.e., the three peaks for Bi_2_TeSe_2_ occur at 70 cm^−1^, 129 cm^−1^ and 171 cm^−1^ and for Bi_2_Se_3_ they move to 71 cm^−1^, 132 cm^−1^ and 174 cm^−1^. This suggests that the stoichiometric ratio of the four compounds has an obvious effect on the vibrational modes. Further understandings about the Raman spectroscopy have been reported in our previous work, ref. 5. To further verify the crystal structure and phase of the Bi_2_Te_*x*_Se_*3−x*_ nanosheets, we performed an X-ray diffractometer (XRD) analysis on the four compounds, as shown in Fig. S1. The XRD pattern can be steadily indexed to the rhombohedral Bi_2_Te_3_ structure (refer to JCPDS Card Number 82–0358, space group: 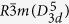)^35^. Comparing the lattice parameters of the four compounds, it is found that the lattice parameters of Bi_2_Te_3_ (a = 4.390 Å, c = 30.460 Å) are slightly larger than those of Bi_2_Te_2_Se (a = 4.303 Å, c = 30.010 Å), Bi_2_TeSe_2_ (a = 4.218 Å, c = 29.240 Å) and Bi_2_Se_3_ (a = 4.140 Å, c = 28.636 Å). This result verifies that the Te atom is really replaced byan Se atom, which leads to the unit cell shrinkage of the Bi_2_Te_*x*_Se_*3−x*_ (x = 0, 1, 2) nanosheets.

The NLO properties of the 2D Bi_2_Te_*x*_Se_*3−x*_ nanosheets were investigated using an open aperture (OA) Z-scan system with femtosecond laser pulses at 532 nm, 800 nm, 1050 nm, and 1550 nm. As the sample was scanned across the focus along the optical axis, the transmitted pulse energies in the presence of the far-field aperture were probed by a detector.

**Figure 3:**
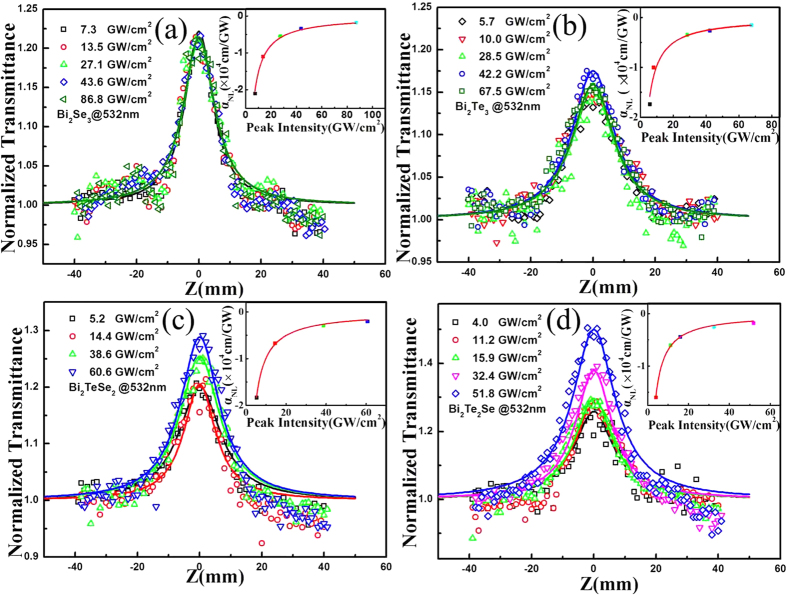
OA Z-scan result of Bi_2_Te_*x*_Se_*3−x*_ for different intensities: (**a**) Bi_2_Se_3_, (**b**) Bi_2_Te_3_, (**c**) Bi_2_TeSe_2_, (**d**) Bi_2_Te_2_Se; Inset: NLO absorption coefficients vary with excitation intensity.

**Figure 4:**
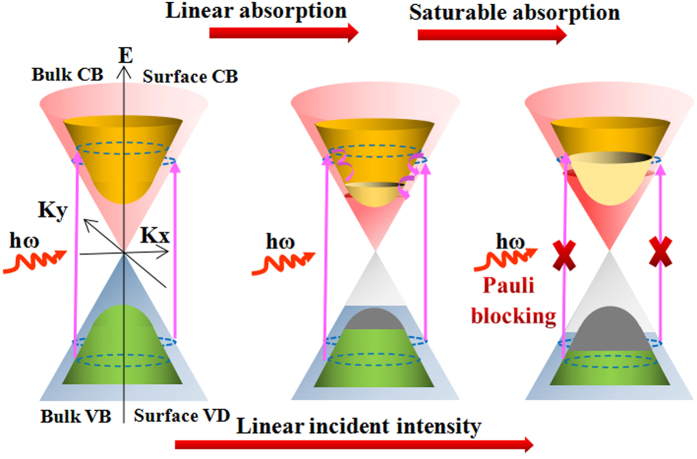
Schematic diagram of optical saturable absorption in Bi_2_Te_*x*_Se_*3−x*_ nanosheets. CB: Conduction band; VB: Valence band.

Figure 3(a–d) show the Z-scan traces of the Bi_2_Te_*x*_Se_*3−x*_ nanosheets that were obtained by a 532-nm laser pulse at different excitation intensities. All of the curves exhibit a “bell shape”, which is induced by the saturable absorption effect (negative NLO absorption). Bi_2_Te_*x*_Se_*3−x*_ nanosheets normally have a narrow band gap (0.15 ~ 0.3 eV)^36^, so it is reasonable to assume that the one-photon induced absorption dominates the NLO process with a photon energy of 0.8 ~ 2.33 eV (λ = 532 nm ~ 1550 nm). The saturable absorption mechanism can be explained as follows: under weak excited light with a photon energy larger than the bulk state bandgap, the electrons in the valence band can be excited to the conduction band and then occupy states in the conduction band, whereas under a high enough intensity of excited light, all the available states in the conduction band are occupied by photo-generated carriers; owing to the Pauli blocking principle, an optical bleaching effect occurs (i.e., saturable absorption). A schematic diagram is shown in Fig. 4.

According to previous reports^27^, for TI nanosheets, the larger the subsurface bulk region, the more sensitive the absorption is to the excitation intensity. For our results in Fig. 3(a,b) on Bi_2_Se_3_ and Bi_2_Te_3_, respectively, the NLO absorption is insensitive to the excitation intensity, which is consistent with the model above. Interestingly, the saturable absorptions of Bi_2_TeSe_2_ and Bi_2_Te_2_Se, in Fig. 3(c,d), respectively, show a greater sensitivity to the excitation intensity. We attribute this to the increased contribution of the bulk conduction state in the nonlinear response. We favour this explanation for two reasons. First, the Bi_2_TeSe_2_ and Bi_2_Te_2_Se samples are tens of nanometres thicker than those of Bi_2_Te_3_ and Bi_2_Se_3_, as shown in Fig. 1(m–p). That means they would possess a much larger bulk region and thereby show much stronger bulk physical properties. Second, the Se atom replacing the Te in Bi_2_Te_3_ can cause disorder (impurities) in the structure, which leads to the symmetry breaking and intrinsic doping. Such a structural change will result in changes in the insulation state and conduction state^13^, which are attributed to the saturable absorption being sensitive to the excitation intensity of the Bi_2_Te_*x*_Se_*3−x*_ (x = 1, 2) nanosheets. With the aim to demonstrate the role of surface effects in the nonlinear response of Bi_2_Te_*x*_Se_*3−x*_ nanosheets, as a representative, we performed an OA Z-scan to measure the nonlinear optical properties of different thickness Bi_2_TeSe_2_ nanosheets. Figure S3(a,b) displays the OA Z-scan results for the two different thickness samples. We can see that all the samples possess a typical saturable absorption property. Different saturable absorption sensitivities to the excitation intensity are observed. The thinner Bi_2_TeSe_2_ nanosheet shows a greater sensitivity to the excitation intensity, which is consistent with the result above. However, it should be noted that this result cannot definitively confirm the role of the surface state in the nonlinear optical properties of the Bi_2_TeSe_2_ nanosheet because it is still challenging to precisely control the crystal morphology in terms of geometry and thickness^5^. Although the thickness of the nanosheets is roughly controlled, its lateral size still varies from several hundred nanometres to several micrometres. This means that the surface-to-volume ratio of the Bi_2_Te_*x*_Se_*3−x*_ nanosheets cannot be precisely controlled. To unambiguously determining the role of the surface state in the large nonlinear coefficients of the Bi_2_Te_*x*_Se_*3−x*_ nanosheets, the exact control of the synthesis is essential, and work is currently underway.

To quantitatively determine the NLO absorption coefficients and identify the corresponding physical mechanisms, we fitted the OA Z-scan data by the NLO absorption model. Based on a spatially and temporally Gaussian pulse, the normalized energy transmittance, T_OA_ (z), is given by^37^,^38^,^39^


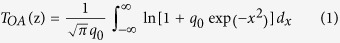


where q_0_ = α_NL_I_0_L_eff_, α_NL_ is the NLO absorption coefficient, L_eff_ = [1−exp (−α_0_L)]/(−α_0_L), α_0_ is the linear optical absorption coefficient and L is the sample path length. By fitting equation (1) to the OA Z-scan curves, the NLO absorption coefficient can be extracted. In Fig. 3(a–d), the solid curves are the fitting results based on the NLO absorption model and agree well with the experimental data. The insets of Fig. 3(a–d) delineate the dependence of the NLO absorption coefficient on the excitation intensity. For the Bi_2_Se_3_ nanosheets, the NLO absorption coefficient increases from −2.1 × 10^4^ cm/GW to −0.34 × 10^4^ cm/GW as the excitation intensity increases from 7.3 GW/cm^2^ to 43.6 GW/cm^2^. It is found that the NLO absorption initially exhibited sustained growth and then reached a plateau as the excitation intensity continued to increase. For the three other Bi_2_Te_*x*_Se_*3−x*_ nanosheets, a similar trend is observed. The mechanism of this trend is unclear. Based on our results, it is reasonable to deduce that there is continuous competition between ground state bleaching and free-carrier absorption (FCA)^37^. We use the energy level diagram shown in Fig. S2 to interpret the evolution of the Bi_2_Te_*x*_Se_*3−x*_ nanosheets’NLO absorption coefficient. As illustrated in Fig. 4, with a high enough excitation intensity, Pauli blocking leads to a saturable absorption in the Bi_2_Te_*x*_Se_*3−x*_ nanosheets. This process corresponds to grounding state bleaching. As the excitation intensity increases, the FCA contributes to the reserve saturable absorption. This FCA process provides a new channel for excited carrier absorption that effectively increases the NLO absorption. The NLO absorption tends to be unchanged owing to the balance between the ground state bleaching and FCA.

To investigate the saturation intensity (I_s_) of the Bi_2_Te_*x*_Se_*3−x*_ nanosheets, a hyperbolic approach saturation numerically model for semiconductors is used, which is expressed as^40^,^41^ 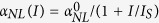, where 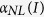
and 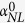 are the intensity-dependent and low-intensity coefficients, respectively. The best fit is shown in the inset of Fig. 3, which indicates that I_S_ ~ 10^9^ W/cm^2^. Table 1 summarizes the saturation intensity I_S_ for Bi_2_Te_*x*_Se_*3−x*_ nanosheets at different wavelengths. The saturation intensity of the as-prepared Bi_2_Te_*x*_Se_*3−x*_ nanosheets sample is consistent with those of the recently reported Bi_2_Se_3_^42^ and Bi_2_TeSe_2_^28^.

**Figure 5:**
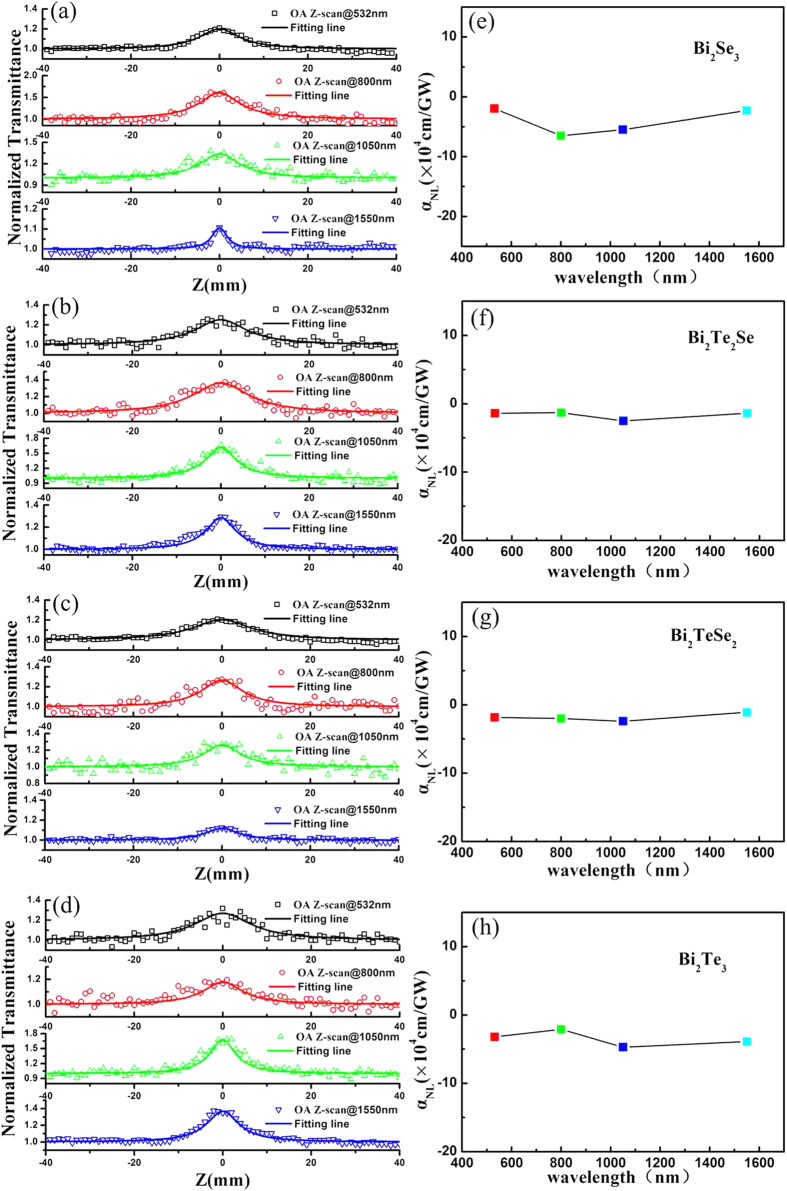
OA Z-scan result of Bi_2_Te_*x*_Se_*3−x*_ nanosheets at different wavelengths (**a**) Bi_2_Se_3_, (**b**) Bi_2_Te_2_Se, (**c**) Bi_2_TeSe_2_, (**d**) Bi_2_Te_3_; (**e**–**h**) NLO absorption coefficients are proportional to wavelength for Bi_2_Se_3_, Bi_2_Te_2_Se, Bi_2_TeSe_2_, and Bi_2_Te_3_, respectively.

With the aim of demonstrating the dependence of the NLO absorption response on the excitation wavelength for Bi_2_Te_*x*_Se_*3−x*_ nanosheets, we performed an OA Z-scan experiment at 800 nm, 1050 nm and 1550 nm. Figure 5(a–d) depicts the Z-scan result of the Bi_2_Te_*x*_Se_*3−x*_ nanosheets at different wavelengths. The saturable absorption process dominates the NLO response induced by a one photon absorption in the Bi_2_Te_*x*_Se_*3−x*_ nanosheets. As noted above, at the considered excitation wavelengths (532 nm, 800 nm, 1050 nm, and 1550 nm), the smallest photon energy 0.8 eV (λ = 1550 nm) may be larger than the band gap of the Bi_2_Te_*x*_Se_*3−x*_ nanosheets. Thus, two-photon and multiphoton absorption may be extremely suppressed during the NLO process at low excitation intensity. The NLO response of the three other Bi_2_Te_*x*_Se_*3−x*_ nanosheets confirmed this phenomenon. In our experiment, for every type of Bi_2_Te_*x*_Se_*3−x*_ nanosheet, the excitation light maintains a similar intensity for all wavelengths.

We extracted the NLO absorption coefficients at different wavelengths, as shown in Fig. 5(e,f). It can be found that the NLO absorption response has no obvious dependence on the excitation wavelength, which means that the NLO absorption is insensitive to the excitation wavelength. In a conventional optical nonlinear enhancement hetero-nanostructure system^43^,^44^, the nonlinear response is sensitive to the excitation wavelength. When the excitation wavelength varies near the surface plasmon resonance band, the field enhancement caused by the surface plasmon resonance increases the incident irradiance. This makes it possible to observe the nonlinear response sensitivity to the excitation wavelength. In the UV-Vis-NIR absorption spectra of the Bi_2_Te_*x*_Se_*3−x*_ nanosheets, in Fig. 2(a), there is no significant surface plasmon resonance absorption in the entire visible and near-infrared range. This indicates that no significant local field enhancement appears as the excitation wavelength changes, which may be attributed to the oscillator strength of the dipole transition being nearly the same under different excitation wavelengths. Additionally, the result reveals that the Bi_2_Te_*x*_Se_*3−x*_ nanosheets present ultra-broadband saturable absorption properties (from the visible-band to the telecommunication C-band), which plays an important role in ultrashort pulsed laser generation.

To understand the other basic NLO properties of the Bi_2_Te_*x*_Se_*3−x*_ nanosheets, we obtained the third-order NLO susceptibility Imχ^(3)^ and the figure of merit FOM of Bi_2_Te_*x*_Se_*3−x*_ nanosheets at different wavelengths. The imaginary part of the third-order NLO susceptibility Imχ^(3)^ can be expressed as^20^ 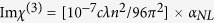, where c is the speed of light in vacuum, λ is the wavelength of the excitation laser, n is the linear refractive index, and α_NL_ is the NLO absorption coefficient. The refractive index n can be calculated from the reflectivity^45^ (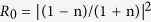, where the reflection spectrum of the Bi_2_Te_*x*_Se_*3−x*_ nanosheets is depicted in Figure S4). The figure of merit was defined to eliminate the discrepancy caused by the linear absorption α_0_: 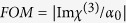. For the Bi_2_Te_3_ nanosheets, the results are demonstrated to be Imχ^(3)^ = (−1.22 ± 0.24) × 10^−8^ esu and FOM = (3.70 ± 0.74) × 10^−13^ esu cm. The values obtained for different wavelengths are listed in Table 1. Compared to previous works, the values of the FOM are approximately two orders of magnitude larger than that of graphene ~5 × 10^−15^ esu cm, graphene oxide ~4.2 × 10^−15^ esu cm^46^, reduced graphene oxide ~0.36 × 10^−15^ esu cm^47^, and MoS_2_/NMP dispersions ~1.06 × 10^−15^ esu cm^20^, one order of magnitude larger than that of multilayer MoS_2_ (25–27 layers) ~1.1 × 10^−14^ esu cm and WS_2_ (18–20 layers) ~2.16 × 10^−14^ esu cm^19^, and close to that of monolayer WS_2_ ~ 1.1 × 10^−13^ esu cm^19^. Our results indicate that the Bi_2_Te_*x*_Se_*3−x*_ nanosheets possess a fascinating NLO response in an ultra-broadband. They can therefore be an excellent potential alternative material for an ON/OFF resonance, saturable absorber.

## Conclusions

In summary, we have performed fundamental and systematic measurements of the NLO response of Bi_2_Te_*x*_Se_*3−x*_ nanosheets using the OA Z-scan technique. Our results demonstrate that the Bi_2_Te_*x*_Se_*3−x*_ nanosheets exhibit ultra-broadband saturable absorption properties and possess fascinating NLO parameters, i.e., α_NL_ ~ −10^4^ cm/GW, Imχ^(3)^ ~ 10^−8^ esu, and FOM ~ 10^−13^ esu cm. Under a ~GW/cm^2^ excitation light intensity, there is insensitive saturable absorption for Bi_2_Se_3_ and Bi_2_Te_3_. Intriguingly, significantly sensitive saturable absorption was observed for Bi_2_Te_2_Se and Bi_2_TeSe_2_, which was ascribed to the change in the bulk region and the atom replacement induced increase of the conduction state. Furthermore, we find that the NLO absorption responses have a weak dependence on the excitation wavelength. This work demonstrates that Bi_2_Te_*x*_Se_*3−x*_ nanosheets are a promising alternative material for saturable absorbers and other nanophotonic devices.

## Additional Information

**How to cite this article**: Wang, Y. *et al*. Ultra-broadband Nonlinear Saturable Absorption for Two-dimensional Bi_2_Te_*x*_Se_*3-x*_ Nanosheets. *Sci. Rep.* **6**, 33070; doi: 10.1038/srep33070 (2016).

## Supplementary Material

Supplementary Information

## Figures and Tables

**Table 1:** Linear and NLO parameters of Bi_2_Te_*x*_Se_*3−x*_ nanosheets measured based on the Z-scan technique.

Sample	λ(nm)	T%	n	α_0_ (×10^4^ cm^−1^)	α_NL_ (×10^4^ cm/GW)	Imχ^(3)^ (×10^−9^ esu)	FOM (×10^−13^ esu cm)	I_S_ (×10^8^ W/cm^2^)
Bi_2_Se_3_	532	0.64	1.039	3.7	−(2.0 ± 0.4)	−(3.6 ± 0.7)	0.96 ± 0.2	19.2 ± 3.8
	800	0.58	1.038	3.7	−(6.5 ± 1.3)	−(17.7 ± 3.5)	4.78 ± 0.2	8.5 ± 1.7
	1050	0.59	1.039	3.3	−(5.5 ± 1.1)	−(19.8 ± 4.0)	6.0 ± 1.2	2.19 ± 0.4
	1550	0.70	1.039	3.3	−(2.3 ± 0.5)	−(12.2 ± 2.4)	3.70 ± 0.7	1.32 ± 0.3
Bi_2_Te_3_	532	0.55	1.044	4.2	−(3.2 ± 0.6)	−(5.8 ± 1.2)	1.38 ± 0.3	10.4 ± 2.0
	800	0.62	1.046	4.3	−(2.1 ± 0.4)	−(5.8 ± 1.2)	1.35 ± 0.2	9.7 ± 1.9
	1050	0.51	1.047	4.6	−(4.7 ± 0.9)	−(17.2 ± 3.4)	3.76 ± 0.8	1.5 ± 0.3
	1550	0.61	1.048	4.3	−(3.9 ± 0.8)	−(21 ± 4.2)	4.9 ± 1.0	5.1 ± 1.0
Bi_2_TeSe_2_	532	0.62	1.046	1.3	−(1.9 ± 0.4)	−(3.4 ± 0.7)	2.68 ± 0.5	4.4 ± 0.9
	800	0.52	1.047	1.2	−(2.0 ± 0.4)	−(5.6 ± 1.1)	4.48 ± 0.9	3.3 ± 0.7
	1050	0.65	1.045	1.2	−(2.4 ± 0.5)	−(8.7 ± 1.7)	7.26 ± 1.5	2.0 ± 0.4
	1550	0.78	1.045	1.1	−(1.1 ± 0.2)	−(5.9 ± 1.2)	5.41 ± 1.1	1.2 ± 0.3
Bi_2_Te_2_Se	532	0.42	1.05	3.4	−(1.4 ± 0.3)	−(2.6 ± 0.5)	0.77 ± 0.2	6.0 ± 1.2
	800	0.40	1.06	3.5	−(1.3 ± 0.3)	−(3.7 ± 0.7)	1.07 ± 0.2	5.2 ± 1.0
	1050	0.56	1.06	3.3	−(2.5 ± 0.5)	−(9.3 ± 1.9)	2.83 ± 0.6	2.0 ± 0.4
	1550	0.57	1.06	3.3	−(1.4 ± 0.3)	−(7.7 ± 1.5)	2.67 ± 0.5	1.6 ± 0.3
