# Supplementary material for: Ultra-broadband Nonlinear Saturable Absorption for Two-dimensional Bi2TexSe3−x Nanosheets
Source: Sci Rep. 2016 Sep 9;6:33070. doi: 10.1038/srep33070 (PMC5016803; doi:10.1038/srep33070)
Supplement: Supplementary Information [file srep33070-s1.pdf]

Ultra-broadband Nonlinear Saturable Absorption for Two-dimensional $Bi_2Te_xSe_{3-x}$ Nanosheets

Yingwei Wang[1‖], ShengLiu[1‖], Jian Yuan[2], Peng Wang[1], Jiazhang Chen[1], Jianbo Li[3], Si Xiao[1*], Qiaoliang Bao[2], Yongli Gao[1,4], &Jun He[1*]

[1] School of Physics and Electronics, Hunan Key Laboratory for Super-micro structure and Ultrafast Process, Central South University, 932 South Lushan Road, Changsha, Hunan 410083, P. R. China;

[2] Institute of Functional Nano and Soft Materials (FUNSOM), Jiangsu Key Laboratory for Carbon-Based Functional Materials and Devices, and Collaborative Innovation Center of Suzhou Nano Science and Technology, Soochow University, Suzhou 215123, P. R. China;

[3] Institute of Mathematics and Physics, Central South University of Forestry and Technology, Changsha 410004, China

[4] Department of Physics and Astronomy, University of Rochester, Rochester, New York 14627, United States;

‖Y. W.Wang and S.Liu contributed equally to this work;

Correspondence and requests for materials should be addressed to S.X. (email: sixiao@csu.edu.cn.) or J.H. (email: junhe@csu.edu.cn)

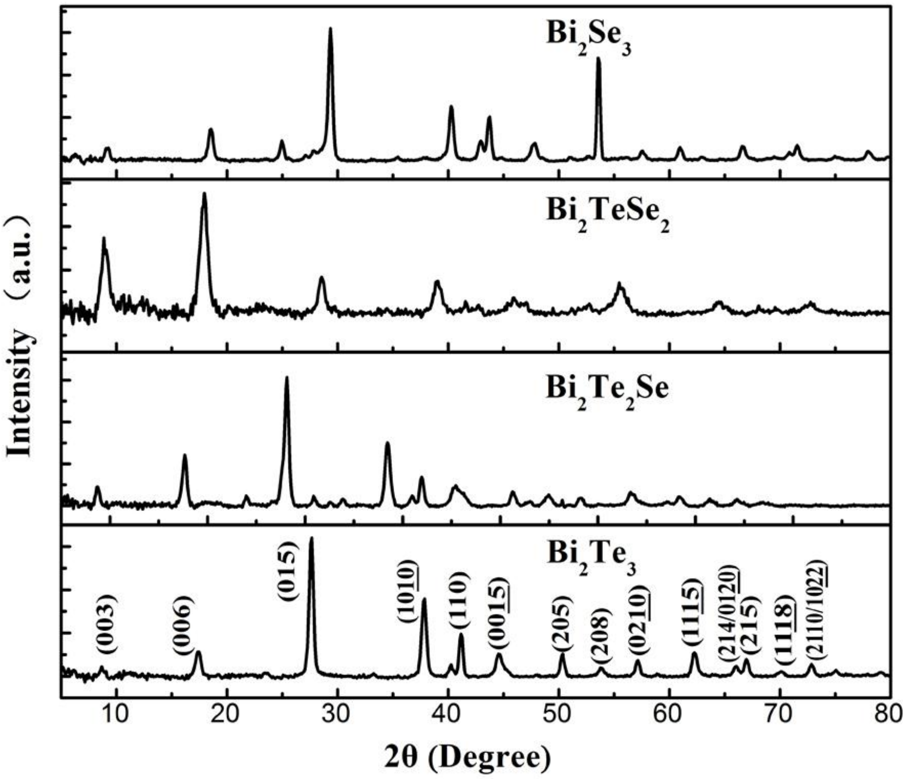

Figure S1. X-ray diffractometer(XRD) patterns of the as-grown $Bi_2Te_xSe_{3-x}$ nanosheets

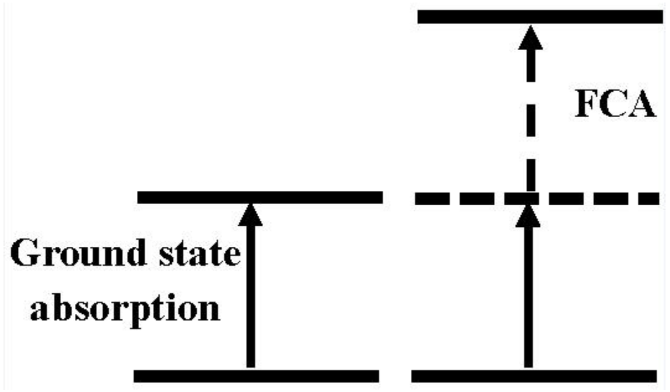

Figure S2. Schematic diagram of main energy level in FCA process.

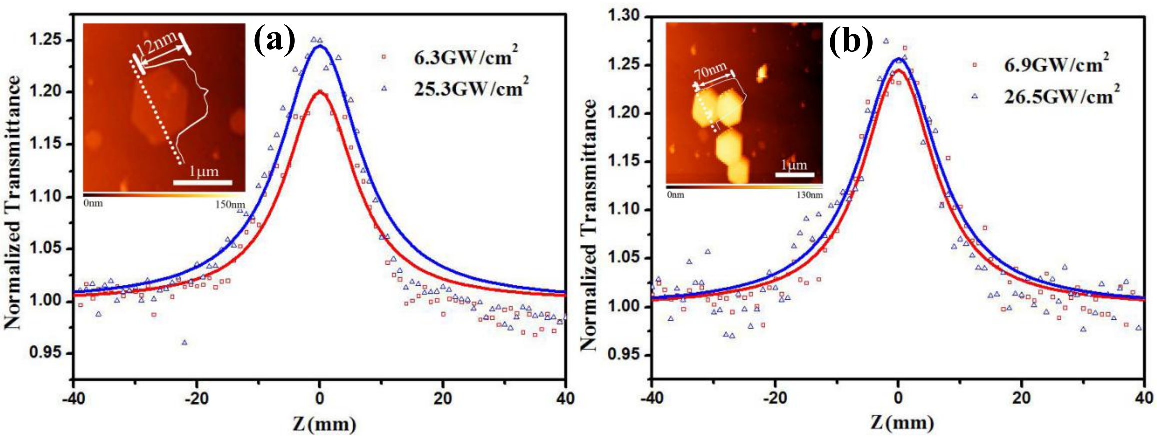

Figure S3. OA Z-scan result of $Bi_2Te_2Se_2$ for different thickness (a)12nm, (b) 70nm, Inset: atomic force microscopy (AFM) images of corresponding sample.

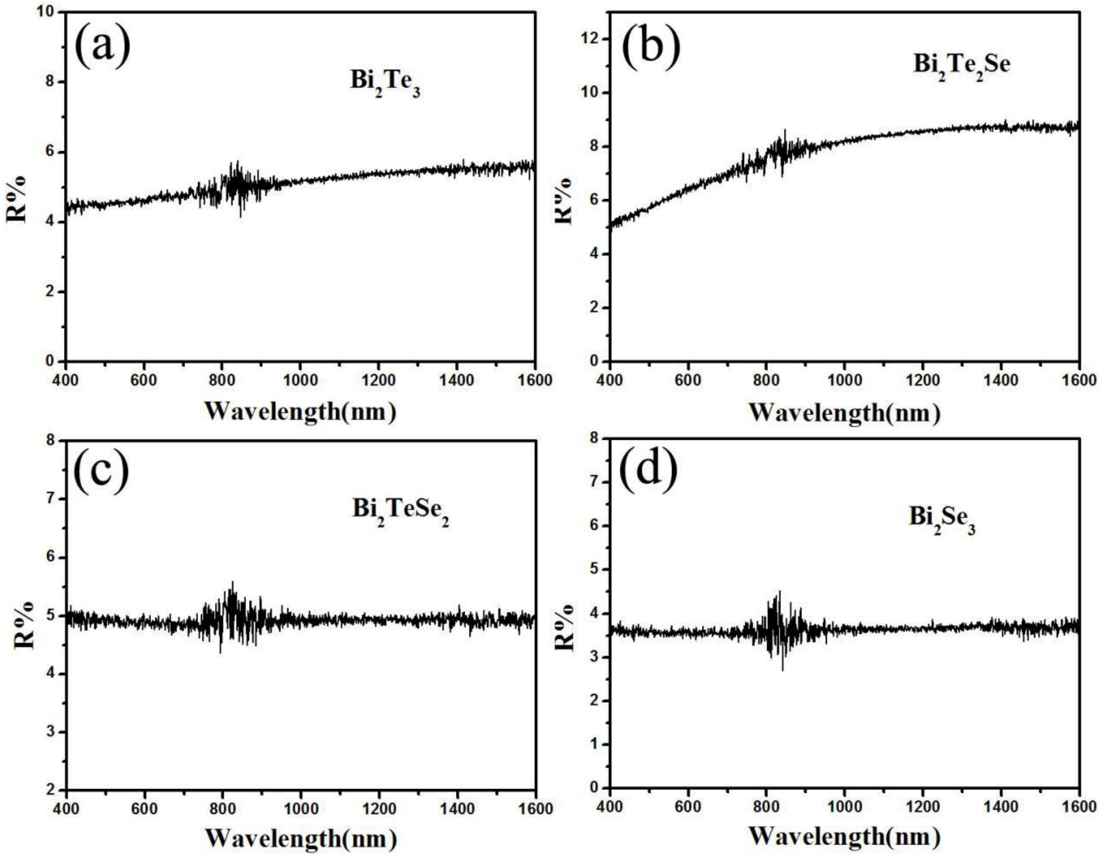

Figure S4. Reflection spectrum of $Bi_2Te_xSe_{3-x}$ nanosheets,(a) Bi_2Te_3; (b)Bi_2Te_2Se; (c)Bi_2TeSe_2; (d)Bi_2Se_3
